# Supplementary material for: Seminoma and Embryonal Carcinoma Footprints Identified by Analysis of Integrated Genome-Wide Epigenetic and Expression Profiles of Germ Cell Cancer Cell Lines
Source: PLoS One. 2014 Jun 2;9(6):e98330. doi: 10.1371/journal.pone.0098330 (PMC4041891; doi:10.1371/journal.pone.0098330)
Supplement: File S1 — ZIP file containing DMRforPairs output for significant regions. Please start from the html files. (ZIP) [file pone.0098330.s008.zip › figures/4959.pdf]

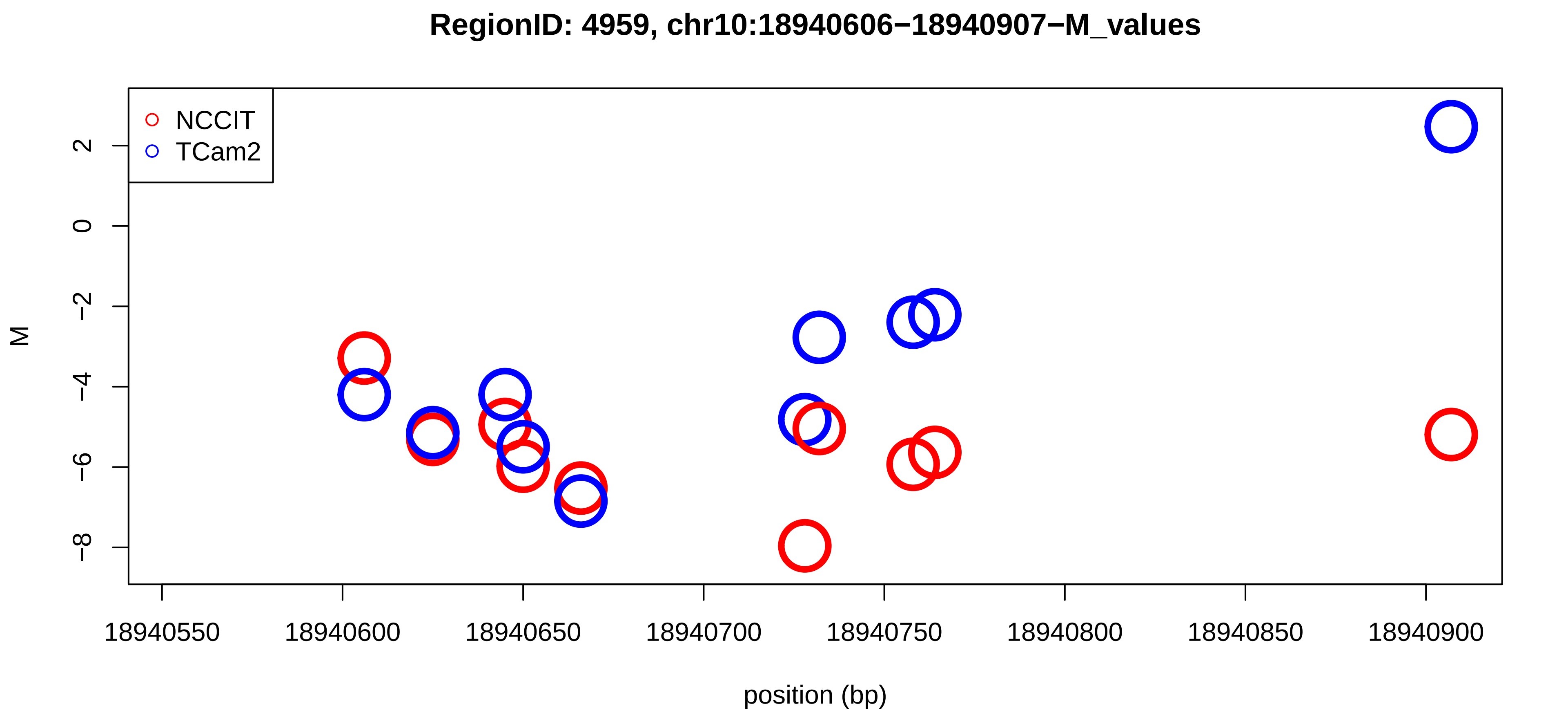
RegionID: 4959, chr10:18940606−18940907−M_values
NCCIT
TCam2
M
2
0
−2
−4
−6
−8
18940550
18940600
18940650
18940700
18940750
18940800
18940850
18940900
position (bp)

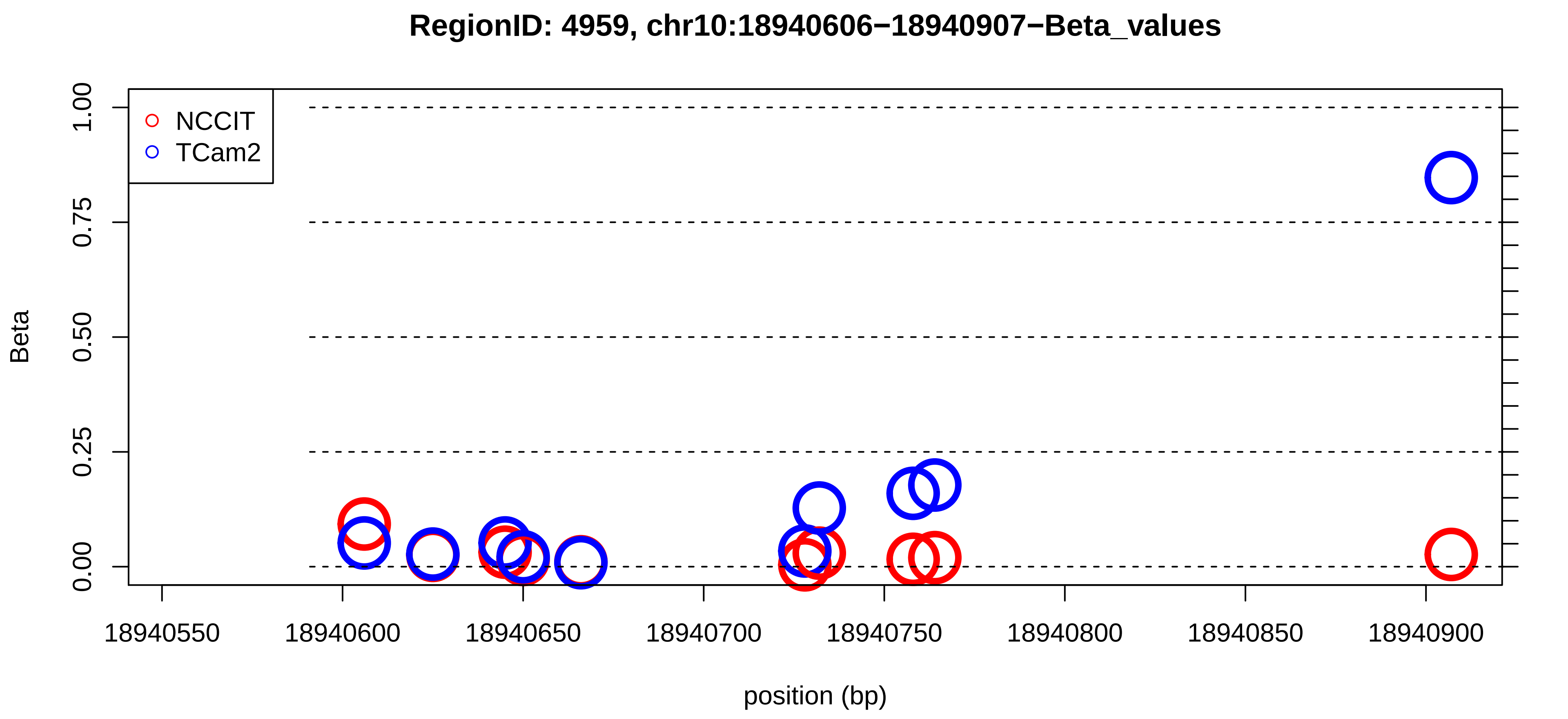
RegionID: 4959, chr10:18940606−18940907−Beta_values
NCCIT
TCam2
Beta
1.00
0.75
0.50
0.25
0.00
18940550
18940600
18940650
18940700
18940750
18940800
18940850
18940900
position (bp)
